# Supplementary material for: Accuracy and Safety Between Robot-Assisted and Conventional Freehand Fluoroscope-Assisted Placement of Pedicle Screws in Thoracolumbar Spine: Meta-Analysis
Source: Medicina (Kaunas). 2025 Apr 9;61(4):690. doi: 10.3390/medicina61040690 (PMC12028692; doi:10.3390/medicina61040690)

**Author/ Year**	**Study type**	**Robot type**	**RS / FS**																	
			**Patients Simple size**	**Screws simple size**	**Gender (male ratio)**	**Age (years)**	**Surgical segment**	**Vertebral level**	**Disease type**	**Operation time (minutes)**	**Blood loss (ml)**	**Mean Radiation dosage (µSv)**	**Accuracy of pedicle screw position**	**Superior facet joint violation**	**Δ VAS**	**Δ ODI**	**Hospital stays (days)**	**Total complications**	**Follow up (months)**	**Overall risk of bias**
Kantelhardt et al., 2011	RCS	Spine Assist	55/57	/	0.83/0.9	62.8/ 63.4	/	/	/	296 /297	/	/	/	/	/	/	10.6 / 14.6	7/22	3	Moderate
Schizas et al., 2012	RCT	Mazor Surgic.	11/23	/	1.2/0.5	65/66	/	D/L/S	DLSD/ LDH/ LSS/ FR	/	/	/	/	/	/	/	/	/	Immediately post op	Low
Roser et al., 2013	RCS	Spine Assist	18/10	72/40	/	/	/	L	LI	/	/	11.03 / 18.9	A:71/39<br>B:1/1<br>C:0/0<br>D:0/0<br>E:0/0	/	/	/	/	/	/	Moderate
Schatlo et al., 2014	RCS	Spine Assist	55/40	/	0.47/0.30	58/52	2.3 ± 0.7 / 2.4 ± 1.2	L	DLSD	205 ± 44 / 189 ± 39	375 ± 263 / 713 ± 455	/	A:204/130<br>B:19/12<br>C:9/10<br>D:4/6<br>E: 2/5	/	/	/	9.8 ± 5.1 / 10.3 ± 5.6	1/2	/	Low
Kim et al., 2015	RCT	Renais.	20/20	80/80	0.55/0.40	64.4 ± 11.9 / 64.7 ± 8.6	/	L	DLSD	217.75 ± 33.90 / 195.00 ± 46.90	/	/	A:76/73<br>B:4/6<br>C:0/1<br>D:0/0<br>E:0/0	/	/	/	/	/	/	Low
Hyun et al., 2017	RCT	Renais.	30/30	/	3.3/0.26	66.8 ± 8.9 / 66.5 ± 8.1	1:20/25<br>2:10/5	/	/	208.5 ± 66.7 / 208.5 ± 62.5	/	55 ± 19 / 122 ± 35	A:127/133.<br>B:3/5<br>C:0/1<br>D:0/1<br>E: 0/0	/	Back: 4.1/3.2<br>Leg: 5.5/5.3	12.4 / 12.3	6.8 ± 2.1 / 9.4 ± 5.4	1/1	16.3	Low
Kim et al., 2017	RCT	Renais.	37/41	158/ 172	0.51/0.54	65.4 ± 10.4 / 66.0 ± 8.6	/	L/S	DLSD/ LS/ LSS	220.1 ± 55.9 / 189.8 ± 45.1	/	/	A:148/158<br>B:9/13<br>C:1/1<br>D:0/0<br>E: 0/0	0:74/69<br>1:0/8<br>2:0/5<br>3: 0/0	Back: 2.5/2.6<br>Leg: 4.1/3.4	29.2 / 27.6	/	0/1	12	Moderate

Keric et al., 2017	RCS	Renais.	66/24	/	0.54/0.56	72.3 ± 11.1 / 68 ± 11.23	/	/	LS	202.55 ± 89.04 / 218.87 ± 85.41	/	/	A:268/43 B:39/46 C:23/25 D:7/3 E: 4/4	/	Back: 3.02/3.0 Leg: 0.19/1.9	9 / 9	13.8 ± 5.6 / 18.1 ± 12.9	15/13	8.4	Low
Laudato et al., 2017	RCS	Mazor Surgic.	36/48	/	/	64/67.5	/	D/L/D	/	/	/	/	-	/	/	/	/	0/0	12	Low
Feng et al., 2019	RCT	TiRobot	40/40	/	0.3/0.325	67.55 ± 6.50 / 67.88 ± 7.34	/	L	DLSD/ LS/	111 ± 33 / 115 ± 26	254.75 ± 115.34 / 356.25 ± 141.00	38.87 ± 11.94 / 47.45 ± 15.75	A:199/206 B:3/18 C:0/1 D:0/0 E:0/0	/	/	/	5.70 ± 1.42 / 5.85 ± 1.33	0/7	/	Low
Han et al., 2019	RCT	TINAVI Medic.	119/115	/	0.48/0.49	54.6 ± 11.3 / 56.1 ± 13.4	/	/	/	149.5 ± 0.8 / 138 ± 48.6	186.0 ± 256.3 / 217 ± 174.3	21.7 ± 11.5 / 70.5 ± 42.0	A:507/503 B:18/43 C:5/27 D:2/8 E: 0/3	/	/	/	4.82 ± 1.5 / 4.95 ± 1.4	0/2	/	Low
Li et al., 2019	RCT	Orthbot	7/10	/	0.43/0.40	/	/	/	DLSD/ LSS	289 ± 87 / 266 ± 92	257 ± 181/ 245 ± 140	/	A:29/39 B:3/10 C:0/1 D:0/0 E:0/0	/	/	/	13.1 ± 2.8 / 12.8 ± 3.2	0/0	/	Moderate
Feng et al., 2020	RCT	TiRobot	40/40	/	0.40/0.38	63.45 ± 4.56 / 64.22 ± 6.19	/	/	LS/ LSS/ LDH/ LI	196.25 ± 62.85 / 230.63 ± 55.06	165.00 ± 102.03 / 237.50 ± 167.47	/	A:167/162 B:3/11 C:0/1 D:0/0 E:0/0	/	Back: 5.83 / 6.05 Leg: 5.13/ 6.18	53.2 / 53	/	1/7	6	Low
Karamian et al., 2021	RCS	Globus Excel. and MMRS	85/177	/	0.447/ 0.446	65.0/ 64.9	1:65/93 2:14/52 3:6/32	/	/	/	/	/	/	/	Back: -2.87 / -2.03 Leg: -3.72 / -2.80	18.29 / 20.87	/	2/4	12	Moderate
Cui et al., 2021	RCT	TianJi Robot System	23/25	/	0.17/0.24	51.3 ± 9.8/ 54.1 ±10.2	/	/	DLSD	135.1 ± 11.2/ 102.2 ± 7.1	173.6 ± 17.9/ 332.1 ± 23.5	/	A:87/85 B:5/15 C-D-E: 0/0	/	Back: 5.3 / 4.6	42.1 / 40.4	7.3 ± 1.8 / 10.0 ± 1.6	0/5	24	Low

Wang et al., 2022	P	TiRobot	61/62	/	0.26/0.34	57.46 ± 8.68 / 57.69 ± 9.15	1:46/45<br>2:15/17	/	DLSD/ LI/ LSS/ LS	160.25 ± 12.13 / 154.35 ± 15.00	78.85 ± 33.52 / 82.90 ± 210.91	/	A:234/196<br>B:32/72<br>C:7/11<br>D:0/2<br>E:1/1	0:246/175<br>1:20/78<br>2:6/24<br>3:2/5	Back: 6.01 / 6.00<br>Leg: 6.24 / 7.12	55.67 / 56.11	4.16 ± 0.71/ 3.94 ± 0.70	/	24	Moderate
Hou et al., 2023	RCS	Renais.	45/56	/	0.33/0.32	14.69 ± 1.93 /14.49 ± 2.01	/	/	/	210.12 ± 11.78 / 179.07 ± 16.60	1063.07 ± 200.04 / 804.56 ± 137.17	/	/	/	/	/	/	1/0	24	Low
Ringel et al., 2012	RCT	Spine Assist	30/30	146/ 152	0.87/ 0.66	68/67	1:17/14<br>2: 13/16	L/S	/	151/132	/	/	A:56/68<br>B:29/25<br>C:11/3<br>D:3/3<br>E:1/1	/	/	/	7/6	/	/	Low

Table S1: Principle characteristics of the included studies in qualitative synthesis. The risk of bias was assessed using the ROBINS-I tool. RS: robotic-assisted surgery; FS: fluoroscopy-assisted surgery; RCT: randomized controlled trial; RCS: retrospective study; P: prospective study; Mazor Surgic.: Mazor Surgical Technologies Ltd; Renais.: Renaissance; TINAVI Medic.: TINAVI Medical Technologies; Globus Excel.: Globus Excelsius GPS robot; MMRS: Medtronic Mazor Robotics Renaissance Guidance System; D: dorsal; L: lumbar; S: sacral; DLSD: degenerative lumbar spinal diseases; LDH: lumbar disc herniation; LI: lumbar instability; LSS: lumbar spinal stenosis; LS: lumbar spondylolisthesis; FR: fractures; ON: oncology; VAS: Visual Analogue Scale; ODI: Oswestry Disability Index; Δ VAS/ODI: difference between pre and post-operative. When present in the original papers, confidence interval (CI) and standard deviation (SD) were inserted.

Supplementary Content
Funnel Plots

Figure S1

A) Publication bias on intraoperative blood loss

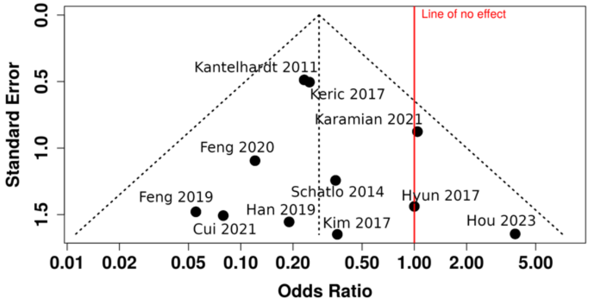

B) Publication bias on total complications

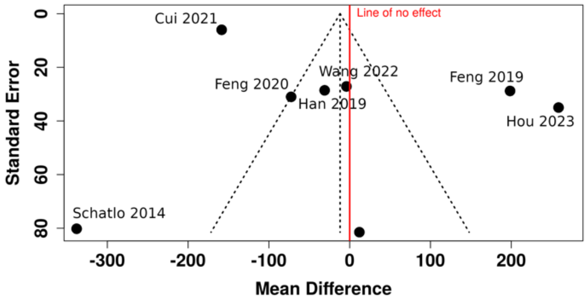

C) Publication bias on hospital stays

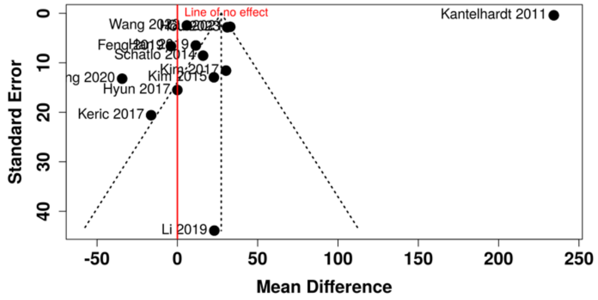

D) Publication bias on operation time

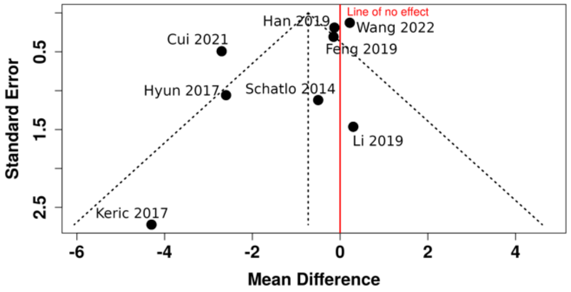

D) Publication bias on radiation dose exposure

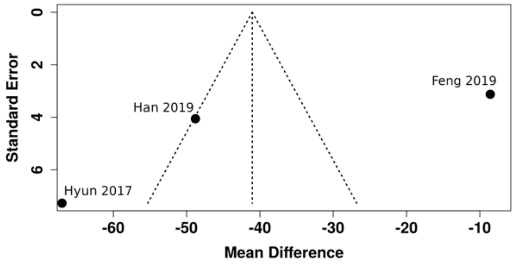

Supplement: Supplementary file 1 [file medicina-61-00690-s001.zip › medicina-3519231-supplementary.pdf]
